# Synthesis of avenanthramides using engineered *Escherichia coli*

**DOI:** 10.1186/s12934-018-0896-9

**Published:** 2018-03-22

**Authors:** Su Jin Lee, Geun Young Sim, Hyunook Kang, Won Seok Yeo, Bong-Gyu Kim, Joong-Hoon Ahn

**Affiliations:** 1 Department of Integrative Bioscience and Biotechnology, Bio/Molecular Informatics Center, Konkuk University, Seoul, 05029 Republic of Korea; 2 Department of Forest Resources, Gyeongnam National University of Science and Technology, 33 Dongjin-ro, Jinju-si, Gyeongsangman-do 52725 South Korea

**Keywords:** Avenanthramides, *Escherichia coli*, Metabolic engineering

## Abstract

**Background:**

Hydroxycinnamoyl anthranilates, also known as avenanthramides (avns), are a group of phenolic alkaloids with anti-inflammatory, antioxidant, anti-itch, anti-irritant, and antiatherogenic activities. Some avenanthramides (avn A–H and avn K) are conjugates of hydroxycinnamic acids (HC), including *p*-coumaric acid, caffeic acid, and ferulic acid, and anthranilate derivatives, including anthranilate, 4-hydroxyanthranilate, and 5-hydroxyanthranilate. Avns are primarily found in oat grain, in which they were originally designated as phytoalexins. Knowledge of the avns biosynthesis pathway has now made it possible to synthesize avns through a genetic engineering strategy, which would help to further elucidate their properties and exploit their beneficial biological activities. The aim of the present study was to synthesize natural avns in *Escherichia coli* to serve as a valuable resource.

**Results:**

We synthesized nine avns in *E. coli*. We first synthesized avn D from glucose in *E. coli* harboring tyrosine ammonia lyase (*TAL*), 4-coumarate:coenzyme A ligase (*4CL*), anthranilate *N*-hydroxycinnamoyl/benzoyltransferase (*HCBT*), and anthranilate synthase (*trpEG*). A *trpD* deletion mutant was used to increase the amount of anthranilate in *E. coli*. After optimizing the incubation temperature and cell density, approximately 317.2 mg/L of avn D was synthesized. Avn E and avn F were then synthesized from avn D, using either *E. coli* harboring *HpaBC* and *SOMT9* or *E. coli* harboring *HapBC* alone, respectively. Avn A and avn G were synthesized by feeding 5-hydroxyanthranilate or 4-hydroxyanthranilate to *E. coli* harboring *TAL*, *4CL*, and *HCBT*. Avn B, avn C, avn H, and avn K were synthesized from avn A or avn G, using the same approach employed for the synthesis of avn E and avn F from avn D.

**Conclusions:**

Using different HCs, nine avns were synthesized, three of which (avn D, avn E, and avn F) were synthesized from glucose in *E. coli*. These diverse avns provide a strategy to synthesize both natural and unnatural avns, setting a foundation for exploring the biological activities of diverse avns.

**Electronic supplementary material:**

The online version of this article (10.1186/s12934-018-0896-9) contains supplementary material, which is available to authorized users.

## Background

Hydroxycinnamic acid amides (HCAAs) are a group of plant secondary metabolites [1] that are categorized into two main types: basic and neutral. Basic amides have primary amines such as spermine, putrescine, and spermidine, whereas neutral amides have aromatic amines, including tyramine, dopamine, anthranilate, and tryptamine [2]. Therefore, the basic amides are water-soluble, whereas the neutral amides are water-insoluble. Initial studies of the physiological functions of these HCAAs revealed allelopathic, antifungal, and antiviral activities [3–5]. Moreover, a recent study demonstrated a role of HCAAs in plant growth and development, responses to abiotic stress, and disease resistance [6]. HCAAs have also shown beneficial effects in humans, including antiviral, melanogenesis, inhibitory, and anticancer activities [7–9]. Among the HCAAs, avenanthramides (anthranilate amides; avns) exhibit anti-genotoxic, anti-inflammatory, anti-fibrotic, anti-itch, and anti-proliferative activities [10–14]. In addition, avns were shown to reduce the risk of atherosclerosis [15]. Oats are a major source of avns [16]. Although various avns and their derivatives have also been synthesized in *Saccharomyces cerevisiae* [17, 18], there is currently no method to synthesize some natural avns (avn A–H and avn K) that are conjugates of hydroxycinnamic acids (HC) such as *p*-coumaric acid, caffeic acid, and ferulic acid and anthranilate derivatives, including anthranilate, 4-hydroxyanthranilate, and 5-hydroxyanthranilate. Therefore, the aim of the current study was to develop a method for the cloning and introduction of genes required for the biosynthesis of these avns in *E. coli*.

To date, several HCAAs have been synthesized by introducing the genes involved in the biosynthetic pathway into *E. coli* [19–22]. In brief, primary amines are synthesized from the amino acids lysine or arginine, as the building blocks of HCAAs [6]. Aromatic amines and HCs are synthesized from phenylalanine, tyrosine, tryptophan, or intermediates of aromatic amino acid biosynthesis. Aromatic amino acid decarboxylases decarboxylate aromatic amino acids, resulting in the synthesis of corresponding amines [23]. HCs are synthesized via a deamination reaction by phenylalanine ammonia lyase or tyrosine ammonia lyase (TAL) and are activated by the attachment of CoA by 4-cinnamate coenzyme A ligase (4CL) [24]. The conjugation reaction between amines and hydroxycinnamoyl-CoAs is mediated by *N*-hydroxycinnamoyl/benzoyltransferase (HCBT), a member of the BAHD acyltransferase family [25, 26], including benzylalcohol *O*-acetyltransferase, anthocyanin *O*-hydroxycinnamoyltransferase, HCBT, and deacetylvindoline 4-*O*-acetyltransferase. Diverse HCBTs have been cloned demonstrating involvement in the synthesis of HC-phenethylamines, HC-tyramines, HC-tryptamines, HC-serotonins, and HC-anthranilates [27–29].

**Fig. 1:**
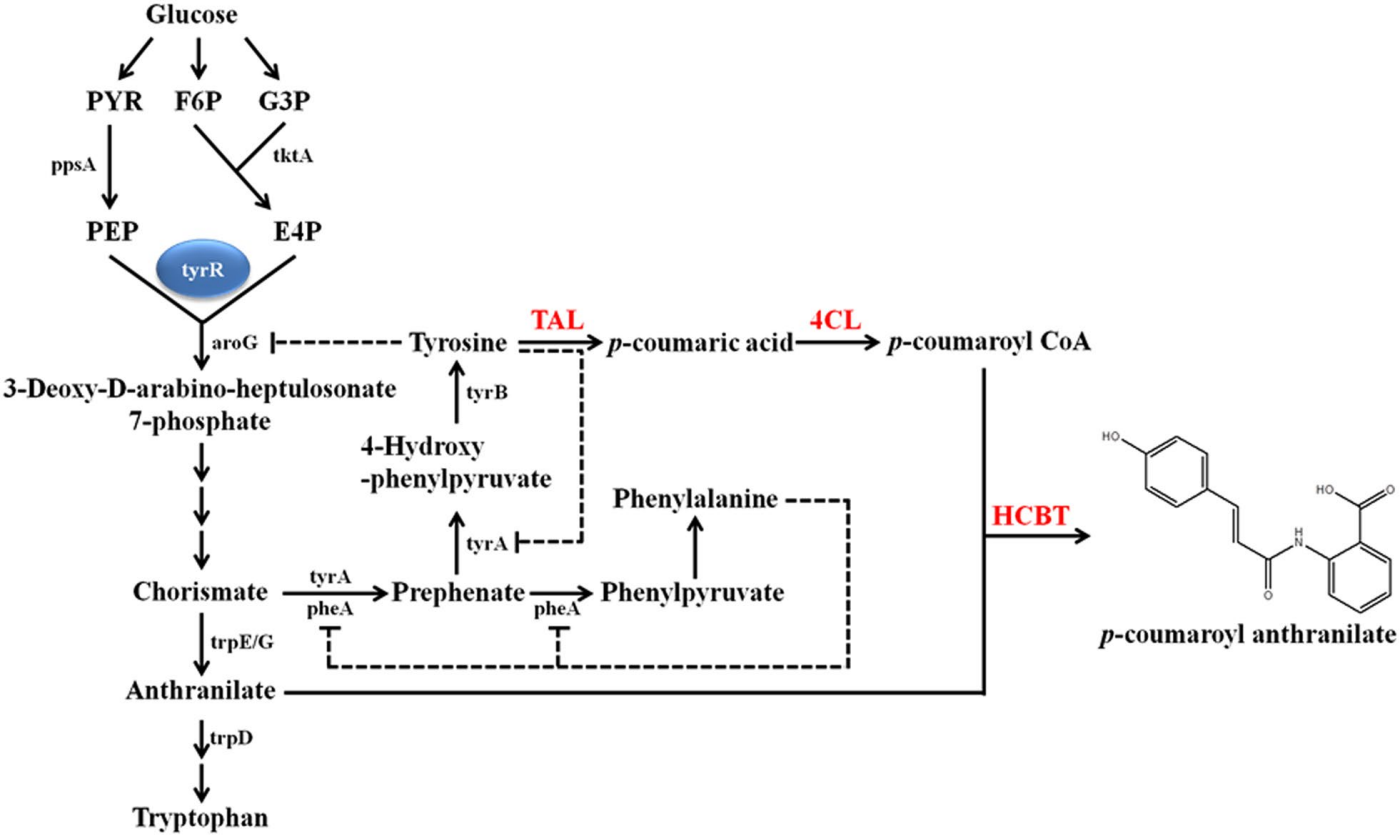
Schematic pathway for the synthesis of *p*-coumaroyl anthranilate in *Escherichia coli*

Based on these known pathways and involved genes, we synthesized nine avns in *E. coli*: avn A–C that use 5-hydroxy anthranilate; avn G, H, and K that use 4-hydroxy anthranilate; and avn D–F consisting of anthranilate (Table 1). In addition, by engineering the shikimic acid pathway through which substrates such as anthranilate and HCs are supplied, avn D was synthesized from glucose (Fig. 1). Avn E and avn F were then synthesized from avn D by modifying the HC portion of avn D. The Avn A series (avn A, B, and C) and avn G series (avn G, H, and K) were synthesized by supplying 5-hydroxyanthranilate and 4-hydroxyanthranilate. This method should serve as a useful resource for further research into the properties and biological activities of avns.

**Table 1:**
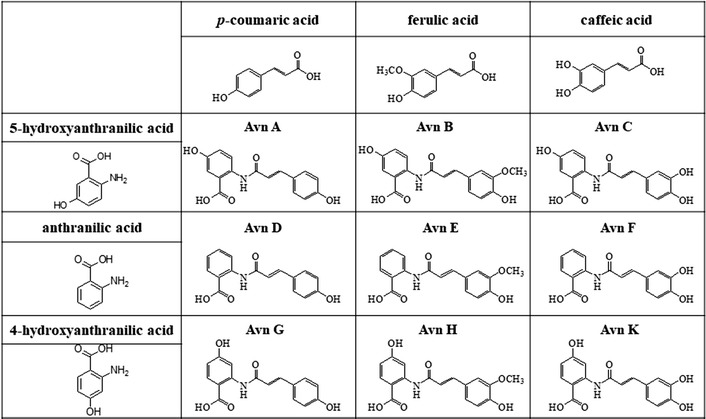
Structure of avenanthramides synthesized in this study

## Results

### Production of various *N*-HC-anthranilates in *E. coli* by feeding HCs

**Fig. 2:**
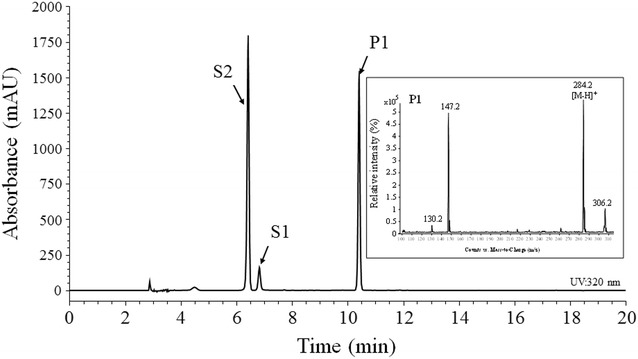
HPLC analysis of the product from *E. coli* harboring BcHCBT. Inset is the molecular mass of the reaction product (A). P1, reaction product; S1, standard anthranilate; S2, *p*-coumaric acid

HC-anthranilate was synthesized from HC-CoA and anthranilate using two genes: *Os4CL*, which converts HC into corresponding HC-CoA, and *HCBT*, which synthesizes HC-anthranilate from HC-CoA and anthranilate. Two *HCBT* genes were tested: one from *D. caryophyllus* (*DcHCBT*) [13] and another from *A. sativa* (*AsHCBT*) [30]. Both genes were subcloned into the *E. coli* expression vector pCDF-Duet along with *Os4CL*. *E. coli* harboring *DcHCBT* and *Os4CL* converted anthranilate and *p*-coumaric acid into *N*-*p*-coumaroyl anthranilate, while *E. coli* harboring *AsHCBT* and *Os4CL* showed approximately 1.5% conversion of these two substrates. Thus, *DcHCBT* was used for further analysis. The molecular mass of the reaction product from *E. coli* harboring *DcHCBT* was 284.2 Da in positive-ion mode, which is the predicted molecular mass of *p*-coumaroyl-anthranilate (Fig. 2). The structure of the compound was confirmed by nuclear magnetic resonance (NMR).

Next, we synthesized additional HC-anthranilate derivatives by feeding strain HA-1 (Table 2) with various HCs and anthranilate. Twelve HCs were used for this purpose. *p*-coumaric acid was found to be the best substrate, followed by *o*-coumaric acid, cinnamic acid, caffeic acid, *m*-coumaric acid, *3*-methoxy cinnamic acid, and ferulic acid (Table 2). The formation of HC-anthranilates was confirmed by mass spectrometry (MS) (data not shown). However, *4*-methoxycinnamic acid, *3*,*4*-dimethoxycinnamic acid, *2*,*4*-dimethoxycinnamic acid, sinapic acid, and *3*,*4*,*5*-trimethoxycinnamic acid were not converted into the corresponding HC-anthranilate under our experimental conditions. As described above, HC-anthranilates were synthesized in two steps: the formation of HC-CoA by Os4CL and conjugation of HC-CoA and anthranilate by HCBT. 4-methoxycinnamic acid was used for the synthesis of *N*-(4-methoxycinnamoyl) tyramine [17], which is also a two-step reaction. This indicates that the attachment of coenzyme A to *4*-methoxycinnamic acid by Os4CL was not problematic, and thus HCBT did not conjugate *4*-methoxycinnamoyl-CoA to anthranilate. For the other substrates tested, including *3*,*4*-dimethoxycinnamic acid, *2*,*4*-dimethoxycinnamic acid, sinapic acid, and *3*,*4*,*5*-trimethoxycinnamic, it was not clear whether the first or second step was responsible for limiting the reaction.

**Table 2:** Conversion of HC and anthranilate into the corresponding N-HC-anthranilate, using strain HA-1

Substrate	Structure	HC-anthranilate^a^ (relative conversion rate %)
*p*-Coumaric acid		100
*o*-Coumaric acid		44.5 ± 2.7
Cinnamic acid		29.1 ± 3.6
Caffeic acid		18.5 ± 2.8
*m*-Coumaric acid		15.0 ± 1.2
3-Methoxycinnamic acid		12.3 ± 2.3
Ferulic acid		6.4 ± 0.8

^a^*E. coli* strain HA-1 was used. Anthranilate and each HC (0.5 mM) was fed and the reaction was carried out at 30 °C for 3 h. The relative conversion rate was calculated by measuring the remaining amount of HC and the product. The best HC was considered as 100. The experiment was carried out in triplicate and the standard deviation was calculated

### Synthesis of anv D, E, and F in *E. coli*

**Fig. 3:**
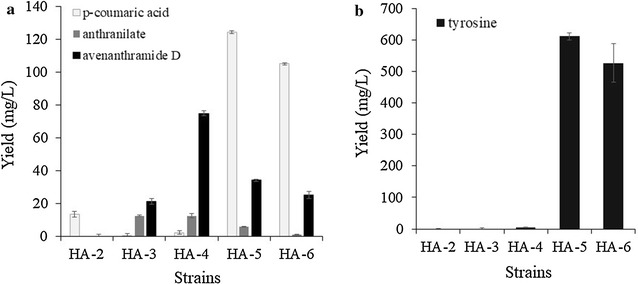
Effect of constructs on the production of avn D in *E. coli.* **a** Production of *p*-coumaric acid, anthranilate and avn D in different strains. **b** Production of tyrosine in different strains

Among the HCs tested as described above, *p*-coumaric acid, caffeic acid, and ferulic acid were further synthesized from glucose using *E. coli* [31]. In addition, anthranilate is an intermediate on aromatic amino acids (tryptophan, tyrosine, and phenylalanine). Thus, we attempted to synthesize *p*-coumaroyl anthranilate (avn D), caffeoyl anthranilate (avn F), and feruloyl anthranilate (avn E). We first synthesized avn D without supplying *p*-coumaric acid and anthranilate. Tyrosine can be used as the substrate for the production of *p*-coumaric acid, and TAL converts tyrosine into *p*-coumaric acid. Anthranilate is an intermediate of tryptophan (Fig. 1). Therefore, *E. coli* cells were transformed with TAL (pA-SeTAL) and pC-Os4CL-HCBT, and the resulting transformant HA-2 was used to examine the synthesis of *p*-coumaroyl anthranilate. However, HA-2 did not synthesize any detectable avn D during the production of *p*-coumaric acid (Fig. 3). It was assumed that the endogenous concentrations of the two substrates, *p*-coumaric acid and anthranilate, were not sufficiently high for the synthesis of *p*-coumaroyl anthranilate. Therefore, three genes, *aroG* (2-dehydro-3-deoxyphosphoheptonate aldolase), *tyrA* (chorismate mutase/prephenate dehydrogenase), and *trpEG* (anthranilate synthase), were overexpressed to increase the intracellular levels of anthranilate and tyrosine. AroG affects anthranilate and tyrosine synthesis because it is the first enzyme in the shikimate pathway [32]. TyrA catalyzes the synthesis of tyrosine from chorismite [32], while TrpEG catalyzes the synthesis of anthranilate from chorismite [33]. We tested the feedback-free version of *aroG* (*aroG*^*fbr*^ and *tyrA*^*fbr*^), both of which are known to increase the intracellular concentration of tyrosine [34]. Using this combination of genes, we prepared four more *E. coli* strains (HA3–6), and the production of avn D was examined in all five *E. coli* strains (HA-2, HA-3, HA-4, HA-5, and HA-6). HA-3, in which *trpEG* was overexpressed, produced avn D, while HA-2, in which *trpEG* was not overexpressed, did not produce any detectable avn D. This indicates that the overexpression of *trpEG* increased the production of anthranilate, which was used to produce avn D. Overexpression of *aroG* and *tyrA* along with *trpEG* (strain HA-4) resulted in 74.9 mg/L avn D, which was much higher than that produced by strain HA-3 (21.4 mg/L) (Fig. 3). Therefore, engineering the pathway for tyrosine and anthranilate increased avn D production. However, the strain overexpressing the feedback-free version of *aroG* and *tyrA* (HA-5; 34.2 mg/L) did not produce more avn D than strain HA-4. In addition, overexpression of *ppsA* and *tktA* (HA-6, 25.0 mg/L), both of which increase phosphoenolpyruvate and erythrose 4-phosphate for the substrate AroG, did not increase avn D production. However, in the strains HA-5 and HS-6, unreacted tyrosine and *p*-coumaric acid were observed (Fig. 3a, b). These results indicate that the metabolic balance between *p*-coumaric acid and anthranilate is important for obtaining higher final yields.

Based on these results, we next optimized the gene combination for the production of avn D. For this purpose, we used an *E. coli* double mutant (BtyrR-trpD), in which *tyrR* and *trpD* were deleted to produce strain HA-7. *tyrR* encodes a transcriptional regulator that regulates the first step of the shikimate pathway and is activated by tyrosine binding [32], and *trpD* encodes anthranilate phosphoribosyl transferase that catalyzes the conversion of anthranilate to *N*-(5-phosphoribosyl) anthranilate [33]. Therefore, deletion of *trpD* increases anthranilate accumulation. Accordingly, it was expected that the *tyrR/trpD* double mutant would increase tyrosine and anthranilate synthesis to ultimately increase avn D production. Indeed, strain HA-7 synthesized 170.8 mg/L of avn D.

**Fig. 4:**
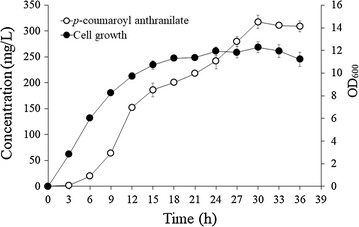
Production of avn D using strain HA-7

The incubation temperature and initial cell density were optimized using this strain. HA-7 was grown at 18, 25, and 30 °C, and the highest production of avn D was observed in cells grown at 25 °C (272.2 mg/L). The yields at 18 and 30 °C were approximately 9.2 and 174.9 mg/L, respectively. The optimal initial cell density was also examined. The cell density was adjusted to OD_600_ = 0.5, 1, 1.5, 2, and 3, and avn D production was examined at 25 °C. The yield of avn D decreased from 307.8 mg/L at OD_600_ = 0.5–197.3 mg/L at OD_600_ = 1.0, 109.7 mg/L at OD_600_ = 1.5, 66.2 mg/L at OD_600_ = 2.0, and 35.8 at OD_600_ = 3.0. Using the optimized incubation temperature and cell density of strain HA-7, the production of *p*-coumaroyl anthranilate was monitored for 36 h. Production of avn D was observed at 3 h and continued to increase until 30 h, at which time the production of avn D was maximized. The yield of avn D at 30 h was 317.4 mg/L (Fig. 4).

**Fig. 5:**
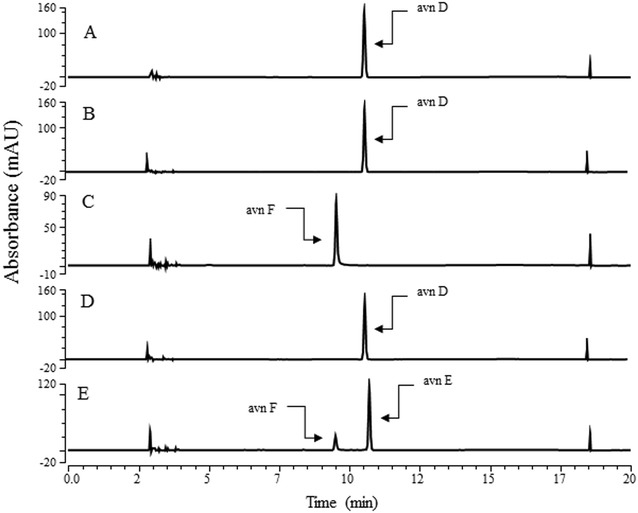
Synthesis of avn F and avn E from avn D. **a** avn D standard indicated as S. **b** Bioconversion of avn D using *E. coli* harboring an empty vector (pGEX5X-3). **c** Bioconversion of avn D using HA-Hpa. **d** Bioconversion of avn D using HA-S. **e** Bioconversion of avn D using both HA-Hpa and HA-S

Next, we synthesized avn F. One additional gene, *HpaBC*, which was reported to convert *p*-coumaric acid into caffeic acid [31] (Additional file 1), was introduced into HA-7 (strain HA-Hpa in Table 3). The resulting strain did not synthesize detectable levels of avn F, but anthranilate and caffeic acid were found in the culture filtrate. A similar phenomenon was previously observed for the synthesis of hydroxysalidroside, in which the introduction of one additional gene in the stepwise synthesis interfered with the whole reaction, resulting in no product formation [35]. Another possible explanation is that HCBT used caffeic acid less effectively than *p*-coumaric acid (Table 3), while the attachment of CoA into either *p*-coumaric acid or caffeic by 4CL was similar [36]. HpaBC is known to convert tyrosine into L-DOPA (3,4-dihydroxyphenyl-l-alanine) [37], which might inhibit the 4CL or HCBT used in this study. Therefore, we used another approach to synthesize avn F by examining whether the strain HA-Hpa could synthesize avn F from avn D, and then both strains HA-Hpa and HA-S would convert avn D into avn F and then avn E. HpaBC was used for the synthesis of caffeic acid from *p*-coumaric acid [31] and SOMT9 was used for the methylation of phenolic compounds having vicinal hydroxy groups [38]. As shown in Fig. 5c, HA-Hpa synthesized a new product and its molecular mass corresponded to that of avn F (Additional file 2). To synthesize avn E from avn D, two *E. coli* transformants (HA-Hpa and HA-S) were fed with avn D and the culture filtrate was analyzed using high-performance liquid chromatography (HPLC). As shown in Fig. 5e, we observed the production of both avn F and avn E, but strain HA-S did not produce any product when fed with avn D (Fig. 5d). This indicated that avn D was converted into avn F by HA-Hpa and then avn F was converted by HA-S.

**Table 3:** Plasmids and strains used in the present study

Plasmids or *E. coli* strain	Relevant properties or genetic marker	Source or reference
Plasmids		
pACYCDuet	P15A ori, Cm^r^	Novagen
pCDFDuet	CloDE13 ori, Str^r^	Novagen
pETDuet	f1 ori, Amp^r^	Novagen
pC-Os4CL-HCBT		
pE-trpEG		
pA-SeTAL	pACYCDuet carrying *TAL* from *Saccharothrix espanaensis*	[42]
pA-aroG-SeTAL-tyrA	pACYCDuet carrying *TAL* from *S. espanaensis*, *aroG* and *tyrA* from *E. coli*	[42]
pA-aroG^fbr^-SeTAL-tyrA^fbr^	pACYCDuet carrying *TAL* from *S. espanaensis*, *aroG*^fbr^, and *tyrA*^fbr^ from *E. coli*	[42]
pA-aroG^fbr^-ppsA-tktA-SeTAL-tyrA^fbr^	pACYCDuet carrying *TAL* from *S. espanaensis*, *aroG*^fbr^, and ppsA, tktA, and *tyrA*^fbr^ from *E. coli*	[42]
pG-HpaBC	pGEX 5X-3 carring *HpaBC* from *E. coli*	[30]
pG-SOMT9	pGEX 5X-3 carring *SOMT9* from soybean	[38]
Strains		
BL21 (DE3)	F^−^ *ompT hsdS*_*B*_ (r_B_^−^ m_B_^−^) *gal dcm lon* (DE3)	Novagen
BtyrR-trpD	BL21 (DE3) *ΔtyrR/ΔtrpD*	This study
HA-1	BL21 harboring pC-Os4CL-HCBT	
HA-2	BL21 harboring pC-Os4CL-HCBT, pA-SeTAL, and pETDuet	This study
HA-3	BL21 harboring pC-Os4CL-HCBT, pA-SeTAL, and pE-trpEG	This study
HA-4	BL21 harboring pC-Os4CL-HCBT, pA-aroG-SeTAL-tyrA, and pE-trpEG	This study
HA-5	BL21 harboring pC-Os4CL-HCBT, pA-aroG^fbr^-SeTAL-tyrA^fbr^, and pE-trpEG	This study
HA-6	BL21 harboring pC-Os4CL-HCBT, pA-aroG^fbr^-ppsA-tktA-SeTAL-tyrA^fbr^, and pE-trpEG	This study
HA-7	BtyrR-trpD harboring pC-Os4CL-HCBT, pA-aroG-SeTAL-tyrA, and pE-trpEG	This study
HA-8	BL21 harboring pC-Os4CL-HCBT, and pA-aroG-SeTAL-tyrA	This study
HA-Hpa	BL21 harboring pG-HpaaBC	This study
HA-S	BL21 harboring pG-SOMT9	This study

Thus, avn F was ultimately synthesized in two steps. Avn D, which was synthesized using strain HA-7, was fed to HA-Hpa at the final concentration of 1121.5 μM (317.4 mg/L). This resulted in the production of 1083.2 μM avn F (324.1 mg/L), giving a conversion rate of approximately 96.5%. Avn E was also synthesized in a stepwise manner. Two *E. coli* transformants (HA-Hpa and HA-S) were mixed with the supernatant of HA-7, which contained avn D. By feeding 1121.5 μM (317.4 mg/L) avn D, 772.4 μM avn F (242.0 mg/L) and 165.4 μM (49.5 mg/L) avn E was synthesized. Approximately 182.9 μM (51.8 mg/L) avn D was not converted to any product and remained in the medium.

### Synthesis of avn A, B, C, G, H, and K

Avn A, B, and C are conjugates of 5-hydroxyanthranilate rather than anthranilate with *p*-coumaric acid, ferulic acid, and caffeic acid, respectively, while avn G, H, and K use 4-hydroxyanthranilate. The gene for the synthesis of 5-hydroxyanthranilate or 4-hydroxyanthranilate from anthranilate is currently unknown. Therefore, we tested whether *E. coli* strain HA-1 could synthesize avn A and avn G from *p*-coumaric acid and 5-hydroxyanthranilate or 4-hydroxyanthranilate, respectively. Both avn A and avn G were synthesized from strain HA-1 supplied with *p*-coumaric acid and 5-hydroxyanthranilate or 4-hydroxyanthranilate, respectively. We also tested other HCs, including caffeic acid and ferulic acid, and found that *p*-coumaric acid served as a substrate, but only small amounts of products were synthesized when caffeic acid and ferulic acid were used. Therefore, we synthesized avn B and avn C from avn A and avn G and synthesized avn H from avn G (see below). Caffeic acid and ferulic acid were not effective for conjugation, even to anthranilate. The other HCs used to test the production of HC-anthranilates did not conjugate to either 5-hydroxyanthranilate or 4-hydroxyanthranilate.

**Fig. 6:**
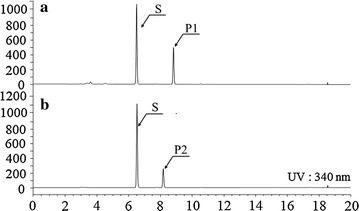
Synthesis of avn G (**a**) by feeding 4-hydroxyanthranilate and *p*-coumaric acid and of avn A (**b**) by feeding 5-hydroxyanthranilate and *p*-coumaric acid. S, *p*-coumaric acid; P1, avn G; P2, avn A

We synthesized avn A and avn G by feeding only 5-hydroxyanthranilate or 4-hydroxyanthranilate using strain HA-8 (Table 3). As shown in Fig. 6, two new products were synthesized with molecular masses corresponding with those of avn A and avn G, respectively (Additional file 2). In addition, the structures of these two products were determined by NMR: 234.1 μM (70.1 mg/L) avn A was synthesized using this strain from 500 μM 5-hydroxyanthranilate, and 299.6 μM (89.6 mg/L) avn G was synthesized from 500 μM 4-hydroxyanthranilate. These results indicated that HA-8 produced the highest levels of avn D, followed by avn G and avn A.

**Fig. 7:**
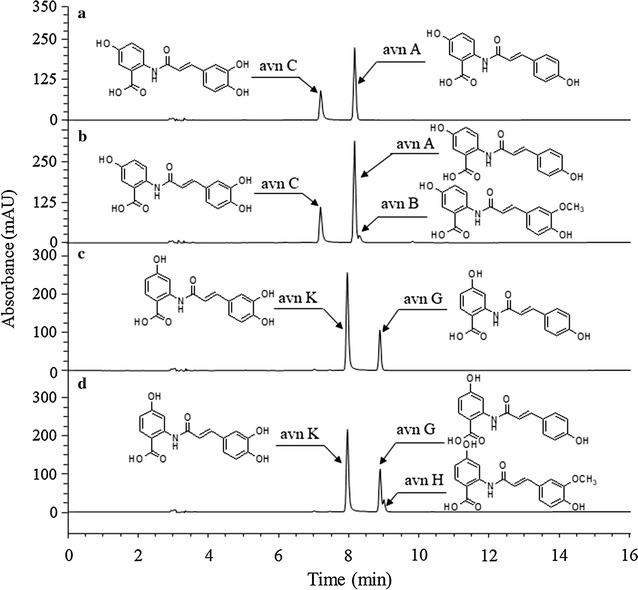
Synthesis of avn C (**a**) and avn B (**b**) from avn A; and synthesis of avn K (**c**) and avn H (**d**) from avn G

Next, we tested whether avn B and avn C were synthesized from avn A while avn H and avn K were synthesized from avn G. The strain HA-Hpa was used to successfully synthesize avn C from avn A and to synthesize avn K from avn G (Fig. 7a, b; Additional file 2). In addition, 31.1 μM avn C was synthesized from 100 μM avn A using HA-Hpa, while 73.3 μM avn K was synthesized from 100 μM avn G. We also synthesized avn B and avn H from avn A and avn G, respectively, using HA-Hpa and HA-S (Fig. 7b, d; Additional file 2). The synthesis of 5.4 μM avn B and 27.0 μM avn C was observed from 100 μM avn A. Finally, 9.2 μM avn H and 60.0 μM avn K were synthesized from 100 μM avn G.

## Discussion

In the present study, we successfully used *E. coli* to synthesize avns found in nature, mainly in oats [27]. As the synthesis of 4-hydroxyanthranilate and 5-hydroxyanthranilate in plants has not been reported, we used commercial 4-hydroxyanthranilate and 5-hydroxyanthranilate to synthesize six avns (avn A, B, C, G, H, and K). Our results showed that a combination of organic and biological synthesis could extend the repertoire of chemicals synthesized.

The key enzyme that increases the final yield of avns in *E. coli* is likely HCBT. HCBT uses anthranilates as acyl acceptors cloned from oat and carnation [13, 30], both of which are major natural sources of avns [27, 39]. However, both these clones showed low activity toward HCs such as caffeic acid, ferulic acid, and hydroxyanthranilates. Oats contain four *HCBT* genes [30]. Thus, further examination of each *HCBT* gene from oats can reveal new genes that can be employed to produce diverse avns.

An additional hydroxyl group in anthranilate influenced the conversion of these hydroxyanthranilates into the corresponding avns. HCBT showed the highest yield of avn D, which was derived from anthranilate and *p*-coumaric acid. HCBT used 5-hydroxyanthranilate more effectively than it used 4-hydroxyanthranilate. HpaBC and SOMT9 showed the highest conversion of avn D into avn F and avn E. Formation of avn C and avn D from avn A using HA-Hpa and HA-S was better than that of avn K and avn H from avn G. This difference may be related to the different transport rate of the substrate, degradation rate, and/or substrate preference of the enzymes (HCBT, HpaBC, and SOMT9).

Two possible in vivo biosynthesis routes for avns are predicted. The first route is the synthesis of all substrates, including HCs and hydroxyanthranilates, and conjugation of these synthesized compound to make diverse avns [40, 41]. The second is the synthesis of basic structures such as avnD and their modified forms produced by hydroxylation and methylation. HCs are synthesized before they undergo the conjugation reaction. Although the specific route used during the in vivo biosynthesis of avns is unknown, we adopted the second route in this study because of the low conversion rate of HCBT.

Avn derivatives have also been synthesized in yeast [29]. However, this study used unnatural substrates such as halogenated anthranilate, halogenated cinnamates, and benzoic acid derivatives along with diverse synthesized derivatives. By contrast, in the present study we focused on the synthesis of avns found in nature. Avns isolated from oats, known as phytoalexins [21], have shown good health benefits, including anti-inflammatory, anti-proliferative, vasodilation, anti-itch, and cytoprotection effects (reviewed by Meydani [27]). Since natural and unnatural avns can both be synthesized using microbial systems, the present strategy opens the door for the exploration of more biological activities using these compounds.

## Conclusions

Avns are natural compounds mainly found in oats and show diverse biological activities. In this study, we cloned the genes necessary for the synthesis of avns and introduced these genes into *E. coli* to synthesize nine avns. In addition, we engineered *E. coli* to provide more substrates for the synthesis of avns. Using this approach, 317.2 mg/L avn D, 49.5 mg/L avn E, and 242.0 mg/L avn F were synthesized from glucose. Avn A, avn G, and their derivatives were also synthesized by feeding of hydroxyanthranilate and stepwise modification of avn A and avn G. The avns synthesized in this study can be used to further explore the biological activities of diverse avns.

## Methods

### Constructs and *E. coli* strains

*Os4CL* was previously cloned in our lab [36] and then subcloned into the *Eco*RI/*Not*I sites of the pCDFDuet1 vector (pC-Os4CL). Hydroxycinnamoyl/benzoyl-CoA:anthranilate HCBT from *Dianthus caryophyllus* for the synthesis of *p*-coumaroyl anthranilate was cloned by polymerase chain reaction (PCR). cDNA was isolated from the leaves of *D. caryophyllus*. PCR was carried out using 5′-AAGAATTCAATGAGTATCCAAATCAAGCAA-3′ (*Eco*RI site is underlined) as a forward primer and 5′-AAGCGGCCGCTTAGAAGTCGTAGAAGTACTT-3′ (NotI site is underlined) as a reverse primer. The resulting PCR product was subcloned into the *Eco*RI/*Not*I sites of pC-Os4CL and the vector was designated as pC-Os4CL-HCBT. HCBT from *Avena sativa* (AsHCBT; GenBank accession number AB076982) was cloned by reverse transcription-PCR using cDNA synthesized from RNA isolated from 2-week-old whole plants as a template. Two primers, 5′-AAGAATTCAATGAAGATCACGGTGCGG-3′ (*Eco*RI site is underlined) and 5′-AAGCGGCCGCTCAGAAGTCGAAGATCATCTTCC-3′ (*Not*I site is underlined), were used, and the PCR product was subcloned into the *Eco*RI/*Not*I sites of pCDF-Duet.

*trpEG* from *E. coli* was amplified by PCR with genomic DNA as a template, using 5′-ATGGATCCCATGCAAACACAAAAACCGACT-3′ (BamHI site is underlined) as a forward primer and 5′-ATCTCGAGTTACAGAATCGGTTGCAGCGTG-3′ (*Xho*I site is underlined) as a reverse primer. The resulting PCR product was subcloned into the *Bam*HI/*Xho*I sites of pET-Duet1 (Novagen, Madison, WI, USA) and named pE-trpEG.

pA-SeTAL, pA-aroG-SeTAL-tyrA, pA-aroG^fbr^-SeTAL-tyrA^fbr^, and pA-aroG^fbr^-ppsA-tktA-SeTAL-tyrA^fbr^ were cloned as reported previously [42].

*HpaBC* and *SOMT9* were cloned as reported previously [31, 38]. The mutant *E. coli* strain BtyrR-trpD was produced using the Quick and Easy Conditional Knockout Kit (Gene Bridges, Heidelberg, Germany), as described by Kim et al. [42]. The plasmids and the strains used in this study are listed in Table 3.

### Synthesis of HC-anthranilate

HCs and anthranilates were purchased from Sigma-Aldrich (St. Louis, MO, USA). HC-anthranilate was synthesized using *E. coli* harboring pC-Os4CL-HCBT. Proteins were induced at 18 °C for 24 h. *E. coli* was harvested and resuspended at OD_600_ = 3.0 in M9 medium containing 2% glucose, 0.5 mM anthranilate, 0.5 mM HC for the synthesis of HC-anthranilate, 100 μg/mL antibiotic(s), and 1 mM isopropyl β-d-1-thiogalactopyranoside. Twelve HC derivatives (*p*-coumaric acid, *m*-coumaric acid, *o*-coumaric acid, caffeic acid, ferulic acid, cinnamic acid, *3*-methoxycinnamic acid, *4*-methoxycinnamic acid, *3*,*4*-dimethoxycinnamic acid, *2*,*4*-dimethoxycinnamic acid, sinapic acid, and *3*,*4*,*5*-trimethoxycinnamic acid) were tested in this study. The culture was incubated at 30 °C for 3 h and extracted with ethyl acetate. After drying the aqueous layer and dissolving this layer in dimethyl sulfoxide, the product was analyzed by HPLC. The mean and standard deviation were calculated from triplicate experiments. The purified *p*-coumaroyl anthranilate was used as a standard for the quantification of all the synthesized avns. For the quantification of anthranilate, *p*-coumaric acid, and tyrosine, these compound were used as the standard.

Metabolites were analyzed using a Thermo Ultimate 3000 HPLC system (Thermo Fisher Scientific, Waltham, MA, USA) equipped with a photo diode array detector and C18 reversed-phase column (4.60 × 250 mm, 3.5-μm particle size; Varian, Palo Alto, CA, USA). The mobile phase consisted of 0.1% formic acid in water and acetonitrile. The program was as follows: 20% acetonitrile at 0 min, 60% acetonitrile at 8 min, 90% acetonitrile at 12 min, 90% acetonitrile at 15 min, 20% acetonitrile at 15.1 min, and 20% at 20 min. The flow rate was 1 min/mL, and ultraviolet detection was performed at 270 and 320 nm.

MS was carried out using matrix-assisted laser desorption/ionization time-of-flight (MALDI-TOF) MS. Samples in MeOH were analyzed by MALDI-TOF MS, using gold nanoparticles as a matrix. An equal volume mixture (1 μL) of the sample and matrix was pipetted onto a stainless steel 384-well target plate (Bruker Daltonics, Billerica, MA, USA), dried in air at room temperature, and analyzed directly by MS using an Autoflex III MALDI-TOF mass spectrometer (Bruker Daltonics) equipped with a smart beam laser as an ionization source. All spectra were acquired at a − 19-kV accelerating voltage and 100-Hz repetition rate for an average of ~ 500 shots. The mass spectra are provided in Additional file 2.

The structure of the reaction product was determined by NMR spectroscopy. The reaction product was extracted with two volumes of ethyl acetate. The organic layer was evaporated and the resulting residue was dissolved in methanol. Anv D was purified by thin-layer chromatography (silica 254F, Merck, Kenilworth, NJ, USA) using chloroform and methanol (22:3) as solvents. The spot containing avn D was collected and extracted with ethyl acetate, and the purity of the product was examined by running HPLC. Two other avns (avn A and avn G) were purified using HPLC. NMR data were as follows: *p*-coumaroyl anthranilate: avn D. ^1^H NMR (Aceton-d6, 400 MHz): δ 6.63 (1H, d, *J *= 15.6 Hz, H-8′), 6.91 (2H, d, *J* = 8.6 Hz, H-3′/5′), 7.16 (1H, ddd, *J* = 1.0, 7.3, 8.0 Hz, H-5), 7.60 (2H, d, *J* = 8.6 Hz, H-2′/6′), 7.62 (1H, m, H-4), 7.66 (1H, d, *J* = 15.6 Hz, H-7′), 8.15 (1H, dd, *J* = 1.5, 8.0 Hz, H-6), 8.90 (1H, dd, *J* = 1.0, 8.5 Hz, H-3). Avn A ^1^H NMR (400 MHz, MeOD-d_4_) δ ppm: 6.50 (d, *J* = 15.6 Hz, H-2), 6.81 (d, *J* = 8.6 Hz, H-6), 7.48 (d, *J* = 8.4 Hz, H-5), 6.90 (dd, *J* = 8.6, 2.8 Hz, H-5′), 7.51 (d, *J* = 2.9 Hz, H-3′), 7.54 (d, *J* = 15.3 Hz, H-3), 8.43 (d, *J* = 8.7 Hz, H-6′). Avn G ^1^H NMR (400 MHz, MeOD-d_4_) δ ppm: 6.52 (dd, *J* = 8.6, 2.8 Hz, H-4′), 6.52 (d, *J* = 15.6 Hz, H-2), 6.82 (d, *J* = 8.6 Hz, H-6), 7.49 (d, *J* = 8.6 Hz, H-5), 7.59 (d, *J* = 15.7 Hz, H-3), 7.96 (d, *J* = 8.7 Hz, H-3′), 8.21 (d, *J* = 2.5 Hz, H-6′). The spectra are shown in Additional file 3.

### Synthesis of *p*-coumaroyl anthranilate and caffeoyl anthranilate

Overnight cultures of *E. coli* HA strains (Table 3) were inoculated into 9 mL of fresh Luria–Bertani medium containing appropriate antibiotics and then cultured to an OD_600_ of 1. The cells were harvested by centrifugation and the cell density was adjusted to OD_600_ = 1 with 10 mL of M9 medium containing 2% glucose, 1% yeast extract, antibiotics, and 1 mM isopropyl β-d-1-thiogalactopyranoside (YM9 medium) in a 100-mL flask. Cells were grown at 30 °C with shaking for 36 h. To analyze product formation, cell growth was monitored by determining the absorbance 600 nm. Culture supernatants were collected, extracted twice with an equal volume of ethyl acetate, and then dried under vacuum. The dried samples were dissolved in dimethyl sulfoxide and analyzed by HPLC on a Varian HPLC system equipped with a photodiode array detector and C18 reverse-phase column (4.60 × 250 mm; 3.5 μm particle size; Agilent Technologies, Santa Clara, CA, USA).

To monitor the production of *p*-coumaroyl anthranilate, strain HA-7 was grown and the cell concentration was adjusted to OD_600_ = 0.5 with YM9 medium followed by incubation at 25 °C. The culture was harvested and analyzed as described above. The mean and standard deviation were calculated from triplicate experiments.

The product was quantified using *p*-coumaroyl anthranilate as a standard. To synthesize avn A and avn G by feeding of only 5-hydroxyanthranilate and 4-hydroxyanthranilate, respectively, strain HA-8 was grown as described above. The reaction was carried out after feeding of 0.5 mM of 4-hydroxyanthranilate or 5-hydroxyanthranilate.

Caffeoyl-anthranilate (avn F) was synthesized in a stepwise manner. *p*-Coumaroyl anthranilate was synthesized using strain HA-7 as described above. *E. coli* harboring *HpaBC* was grown as described above and harvested by centrifugation. The cells were resuspended with the supernatant from HA-7 at a final cell density of OD_600_ = 3.0. To synthesize avn E, two *E. coli* strains (*E. coli* harboring HpaBC and *E. coli* harboring SOMT9) were resuspended with the culture supernatant of HA-7 at a cell density of OD_600_ = 3.0. The resulting mixture was incubated at 30 °C for 30 h with shaking at 180 rpm and analyzed by HPLC after ethyl acetate extraction. To synthesize avn C using *E. coli* harboring HpaBC and to synthesize avn B using *E. coli* harboring HpaBC and *E. coli* harboring SOMT9, the initial cell density was OD_600_ = 3.0 and 100 μM of avn A was used. Avn K and avn H were synthesized from avn G by the same method as described for synthesizing avn C and avn B from avn A.

## Additional files

**Additional file 1.** Bioconversion of *p*-coumaric acid and caffeic acid.

**Additional file 2.** Mass spectra of avn A (a), avn G (b), avn D (c), avn B (d), avn H (e), avn E (f), avn C (g), avn K (h), and avn F (i).

**Additional file 3.** Proton NMR spectra of avn A (a), avn D (b), and avn G (c).

## Abbreviations

avn: avenanthramide
HPLC: high-performance liquid chromatography
NMR: magnetic resonance spectroscopy

## References

[1] Martin-Tanguy J (1985). The occurrence and possible function of hydroxycinnamic acid amides in plants. Plant Growth Regul.

[2] Facchini PJ, Hagel J, Zulak KG (2002). Hydroxycinnamic acid amide metabolism: physiology and biochemistry. Can J Bot.

[3] Yoshihara T, Takamatsue S, Sakamura S (1978). Three new phenolic amides from roots of eggplant (*Solanum melongena* L.). Agric Biol Chem.

[4] Stoessl A, Unwin CH (1969). The antifungal factors in barley. V. Antifungal activity of the hordatines. Can J Bot.

[5] Martin-Tanguy J, Cabanne F, Perdrizet E, Martin C (1978). The distribution of hydroxycinnamic acid amides in flowering plants. Phytochemistry.

[6] Bassard JE, Ullmann P, Bernier F, Werck-Reichhart B (2010). Phenolamides: bridging polyamides to the phenolic metabolism. Phytochemitsty.

[7] Okombi S, Rival D, Bonnet S, Mariotte A-M, Perrier E, Boumendje A (2006). Analogues of *N*-hydroxycinnamoylphenalkylamides as inhibitors of human melanocyte-tyrosinase. Bioorg Med Chem Lett.

[8] Park JB, Schoene N (2003). *N*-Caffeoyltyramine arrests growth of U937 and Jurkat cells by inhibiting protein tyrosine phosphorylation and inducing caspase-3. Cancer Lett.

[9] Nesterenko V, Putt KS, Hergenrother PJ (2003). Identification from a combinatorial library of a small molecule that selectively induces apoptosis in cancer cells. J Am Chem Soc.

[10] Koening RT, Dickman JR, Wise ML, Ji LL (2011). Avenanthramides are bioavailable and accumulate in hepatic, cardiac, and skeletal muscle tissue following oral gavage in rats. J Agri Food Chem.

[11] Kurtz ES, Wallo W (2007). Colloidal oatmeal: history, chemistry and clinical properties. J Drugs Dermatol.

[12] Lee-Manion AM, Price RK, Strain JJ, Dimberg LH, Sunnerheim K, Welch RW (2009). In vitro antioxidant activity and antigenotoxic affects of avenanthramides and related compounds. J Agric Food Chem.

[13] Singh R, De S, Belkheir A (2013). Avena sativa (oat), a potential neutraceutical and therapeutic agent: an overview. Ctri Rev Food Sci Nutr.

[14] Zammit SC, Cox AJ, Goe RM, Zhang Y, Gilbert RE, Krum H, Kelly DJ, Williams SJ (2009). Evaluation and optimization of antifibrotic activity of cinnamoyl anthranilates. Bioorg Med Chem Lett.

[15] Nie L, Wise ML, Peterson DM, Meydani M (2006). Avenanthramide, a polyphenol from oats, inhibits vascular smooth muscle cell proliferation and enhances nitric oxide production. Atherosclerosis.

[16] Meydani M (2009). Potential health benefits of avenanthramides of oats. Nutr Rev.

[17] Eudes A, Baidoo EE, Yang F, Burd H, Hadi MZ, Collins FW, Keasling JD, Loqué D (2011). Production of tranilast [*N*-(3′,4′-dimethoxycinnamoyl)-anthranilic acid] and its analogs in yeast *Saccharomyces cerevisiae*. Appl Microbiol Biotechnol.

[18] Eudes A, Teixeira Benites V, Wang G, Baidoo EE, Lee TS, Keasling JD, Loqué D (2015). Precursor-directed combinatorial biosynthesis of cinnamoyl, dihydrocinnamoyl, and benzoyl anthranilates in *Saccharomyces cerevisiae*. PLoS ONE.

[19] Eudes A, Juminaga D, Baidoo EE, Collins FW, Keasling JD, Loqué D (2013). Production of hydroxycinnamoyl anthranilates from glucose in *Escherichia coli*. Microb Cell Fact.

[20] Kang K, Back K (2009). Production of phenylpropanoid amides in recombinant *Escherichia coli*. Metab Eng.

[21] Park M, Kang K, Park S, Kim YS, Ha SH, Lee SW, Ahn MJ, Bae JM, Back K (2008). Expression of serotonin derivative synthetic genes on a single self-processing polypeptide and the production of serotonin derivatives in microbes. Appl Microbiol Biotechnol.

[22] Sim GY, Yang SM, Kim BG, Ahn JH (2015). Bacterial synthesis of *N*-hydroxycinnamoyl phenethylamines and tyramines. Microb Cell Fact.

[23] Facchini PJ, Huber-Allanach KL, Tari LW (2000). Plant aromatic l-amino acid decarboxylases: evolution, biochemistry, regulation, and metabolic engineering applications. Phytochemistry.

[24] Vogt T (2010). Phenylpropanoid biosynthesis. Mol Plant.

[25] St-Pierre B, De Luca V (2000). Evolution of acyltransferase genes: origin and diversification of the BAHD superfamily of acyltransferases involved in secondary metabolism. Recent Adv Phytochem.

[26] D’Auria JC (2006). Acyltransferases in plants: a good time to be BAHD. Curr Opin Plant Biol.

[27] Back K, Jang SM, Lee BC, Schmidt A, Strack D, Kim KM (2001). Cloning and characterization of a hydroxycinnamoyl-CoA:tyramine *N*-(hydroxycinnamoyl)transferase induced in response to UV-C and wounding from *Capsicum annuum*. Plant Cell Physiol.

[28] Jang SM, Ishihara A, Back K (2004). Production of coumaroylserotonin and feruloylserotonin in transgenic rice expressing pepper hydroxycinnamoyl-coenzyme A:serotonin *N*-(hydroxycinnamoyl)transferase. Plant Physiol.

[29] Yang Q, Grimmig B, Matern U (1988). Anthranilate *N*-hydroxycinnamoyl/benzoyltransferase gene from carnation: rapid elicitation of transcription and promoter analysis. Plant Mol Biol.

[30] Yang Q, Trinh HX, Imai S, Ishihara A, Zhang L, Nakayashiki H, Tosa Y, Mayama S (2004). Analysis of the involvement of hydroxyanthranilate hydroxycinnamoyltransferase and caffeoyl-CoA 3-*O*-methyltransferase in phytoalexin biosynthesis in oat. Mol Plant Microbe Interact.

[31] An DG, Cha MN, Nadarajan SP, Kim BG, Ahn JH (2016). Bacterial synthesis of four hydroxycinnamic acids. Appl Biol Chem.

[32] Sprenger GA (2007). From scratch value; engineering *Escherichia coli* wild type cells to the production of l-phenylalanine and other fine chemicals derived from chorismite. Appl Microbial Biotechnol.

[33] Ikeda M (2006). Towards bacterial strains overproducing l-tryptophan and other aromatics by metabolic engineering. Appl Microbiol Biotechnol.

[34] Lütke-Eversloh T, Stephanopoulos G (2007). l-Tyrosine production by deregulated strains of *Escherichia coli*. Appl Microbiol Biotechnol.

[35] Chung D, Kim SY, Ahn JH (2017). Production of three phenylethanoids, tyrosol, hydroxytyrosol, and salidroside, using plant genes expressing in *Escherichia coli*. Sci Rep.

[36] Lee YJ, Jeon Y, Lee JS, Kim BG, Lee CH, Ahn JH (2007). Enzymatic synthesis of phenolic CoAs using 4-coumarate:coenzyme A ligase (4CL) from rice. Bull Korean Chem Soc.

[37] Wei T, Cheng BY, Liu JZ (2016). Genome engineering *Escherichia coli* for l-DOPA overproduction from glucose. Sci Rep.

[38] Park SH, Kim BG, Lee SH, Lim Y, Cheong Y, Ahn J-H (2007). Molecular modeling and site directed mutagenesis of the *O*-methyltransferase, SOMT-9 reveals amino acids important for its reaction and regioselectivity. Bull Korean Chem Soc.

[39] Yang Q, Reinhard K, Schiltz E, Matern U (1997). Characterization and heterologous expression of hydroxycinnamoyl/benzoyl-CoA:anthranilate *N*-hydroxycinnamoyl/benzoyltransferase from elicited cell cultures of carnation *Dianthus caryophyllus* L. Plant Mol Biol.

[40] Gutierrez-Gonzalez JJ, Tu ZJ, Garvin DF (2013). Analysis and annotation of the hexaploid oat seed transcriptome. BMC Genom.

[41] Okazaki Y, Isobe T, Iwata Y, Matsukawa T, Mstsuda F, Miyagawa H, Ishihara A, Nishioka T, Iwamura H (2004). Metabolism of avenanthramide phytoalexins in oats. Plant J.

[42] Kim MJ, Kim BG, Ahn JH (2013). Biosynthesis of bioactive *O*-methylated flavonoids in *Escherichia coli*. Appl Microbiol Biotechnol.

