# Supplementary material for: Synthesis of avenanthramides using engineered Escherichia coli
Source: Microb Cell Fact. 2018 Mar 22;17:46. doi: 10.1186/s12934-018-0896-9 (PMC5863376; doi:10.1186/s12934-018-0896-9)
Supplement: Supplementary file 1 — Additional file 1. Bioconversion of p-coumaric acid and caffeic acid. [file 12934_2018_896_MOESM1_ESM.docx]

Figure. Bioconversion of *p*-coumaric acid. A, standard *p*-coumaric acid; B, standard caffeic acid; C, standard ferulic acid; D, bioconversion of *p*-coumaric acid using *E. coli* harboring an empty vector (pGEX 5X-3); E. bioconversion of *p*-coumaric acid using HA-Hpa; F, bioconversion of *p*-coumaric acid using HA-S; bioconversion of *p*-coumaric acid using both HA-Hpa and HA-S.

Figure. Bioconversion of caffeic acid. A, standard caffeic acid; B, standard ferulic acid; C, bioconversion of *p*-coumaric acid using *E. coli* harboring an empty vector (pGEX 5X-3); D. bioconversion of *p*-coumaric acid using HA-Hpa; E, bioconversion of *p*-coumaric acid using HA-S
