# Supplementary figures and images for: Synthesis of avenanthramides using engineered Escherichia coli
Source: Microb Cell Fact. 2018 Mar 22;17:46. doi: 10.1186/s12934-018-0896-9 (PMC5863376; doi:10.1186/s12934-018-0896-9)

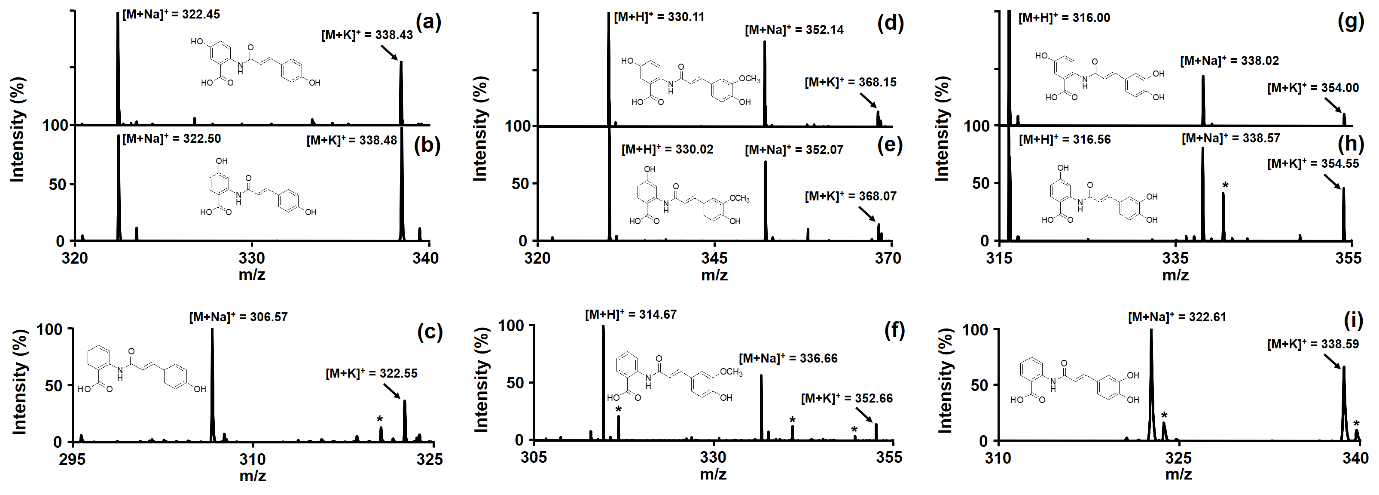


Mass spectra of avn A (a), avn G (b), avn D (c), avm B (d), avn H (e), avn E (f), avn C (g), avn K (h), and avn F (i).

Supplement: Supplementary file 2 — Additional file 2. Mass spectra of avn A (a), avn G (b), avn D (c), avn B (d), avn H (e), avn E (f), avn C (g), avn K (h), and avn F (i). [file 12934_2018_896_MOESM2_ESM.docx]
