# Supplementary material for: Synthesis of avenanthramides using engineered Escherichia coli
Source: Microb Cell Fact. 2018 Mar 22;17:46. doi: 10.1186/s12934-018-0896-9 (PMC5863376; doi:10.1186/s12934-018-0896-9)
Supplement: Supplementary file 3 — Additional file 3. Proton NMR spectra of avn A (a), avn D (b), and avn G (c). [file 12934_2018_896_MOESM3_ESM.docx]

a


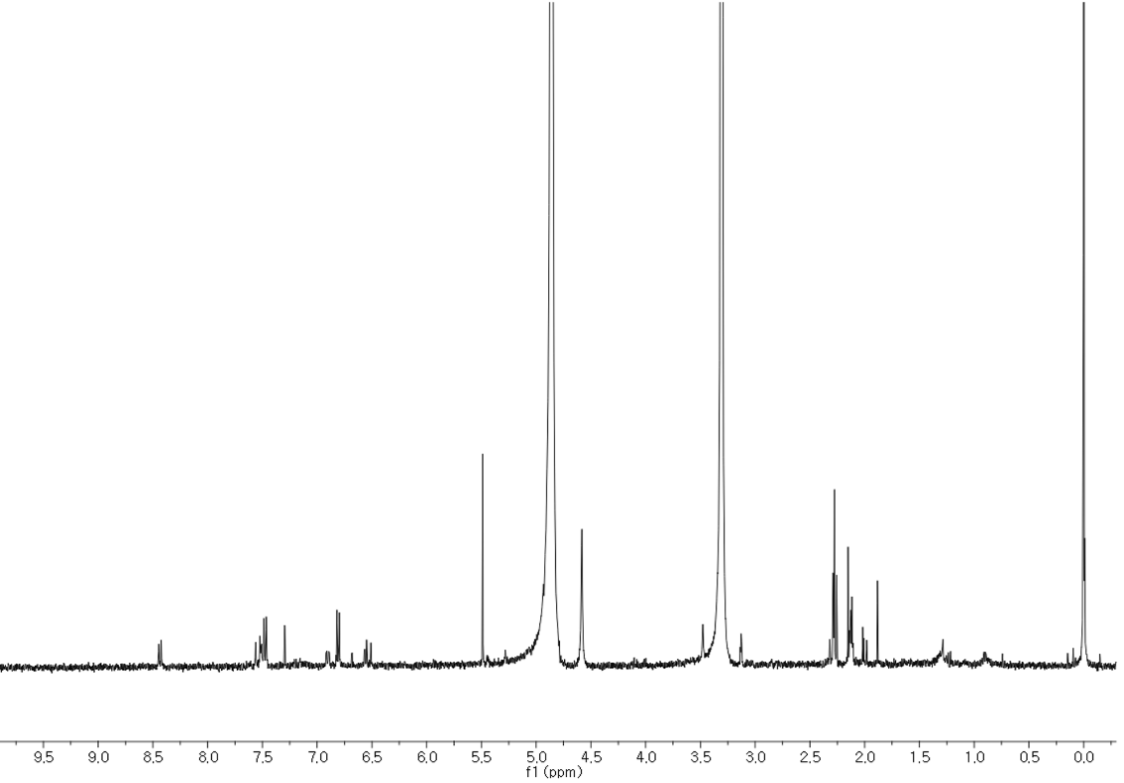


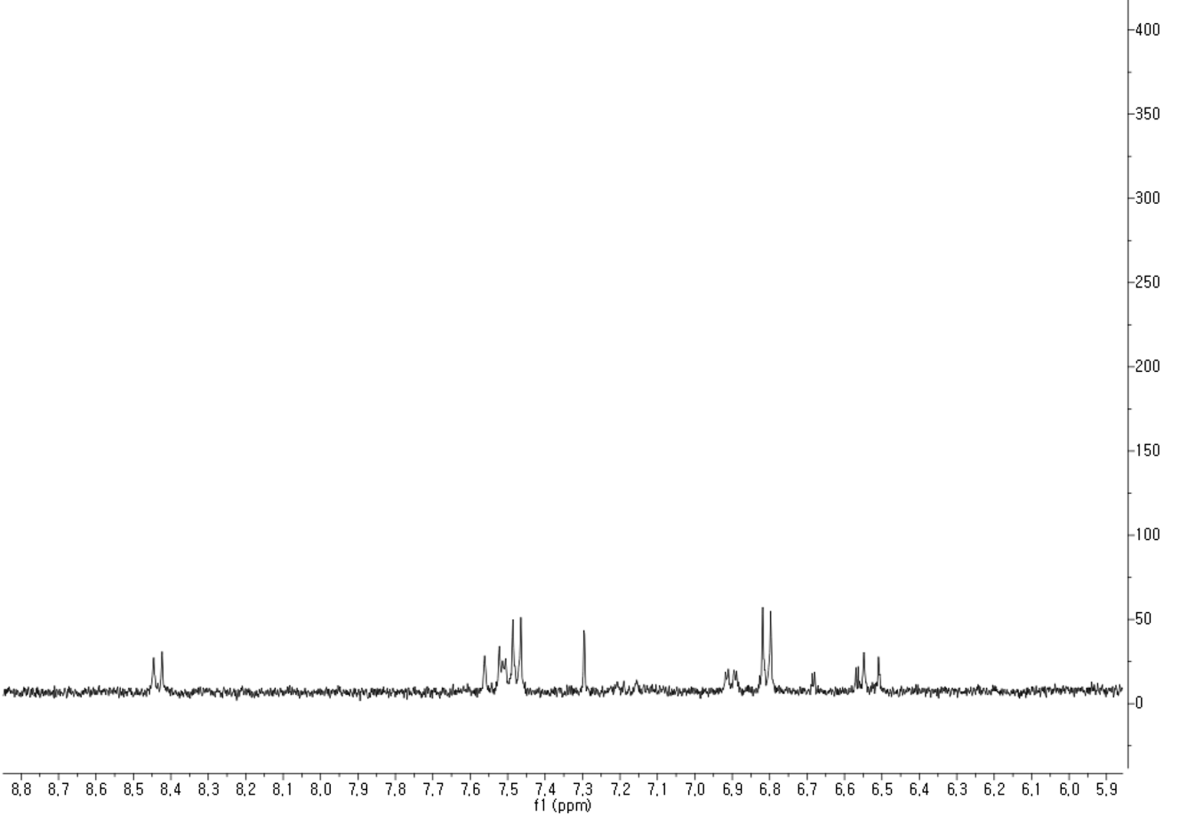


b


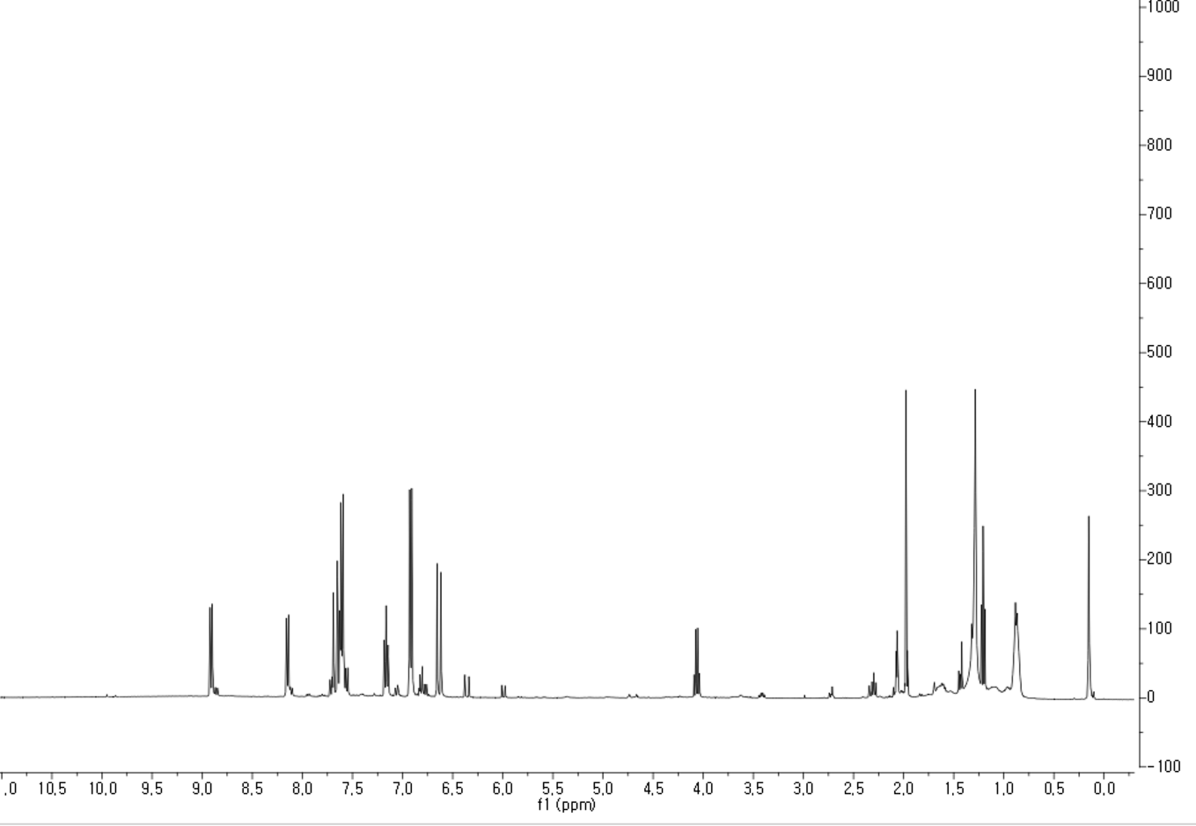


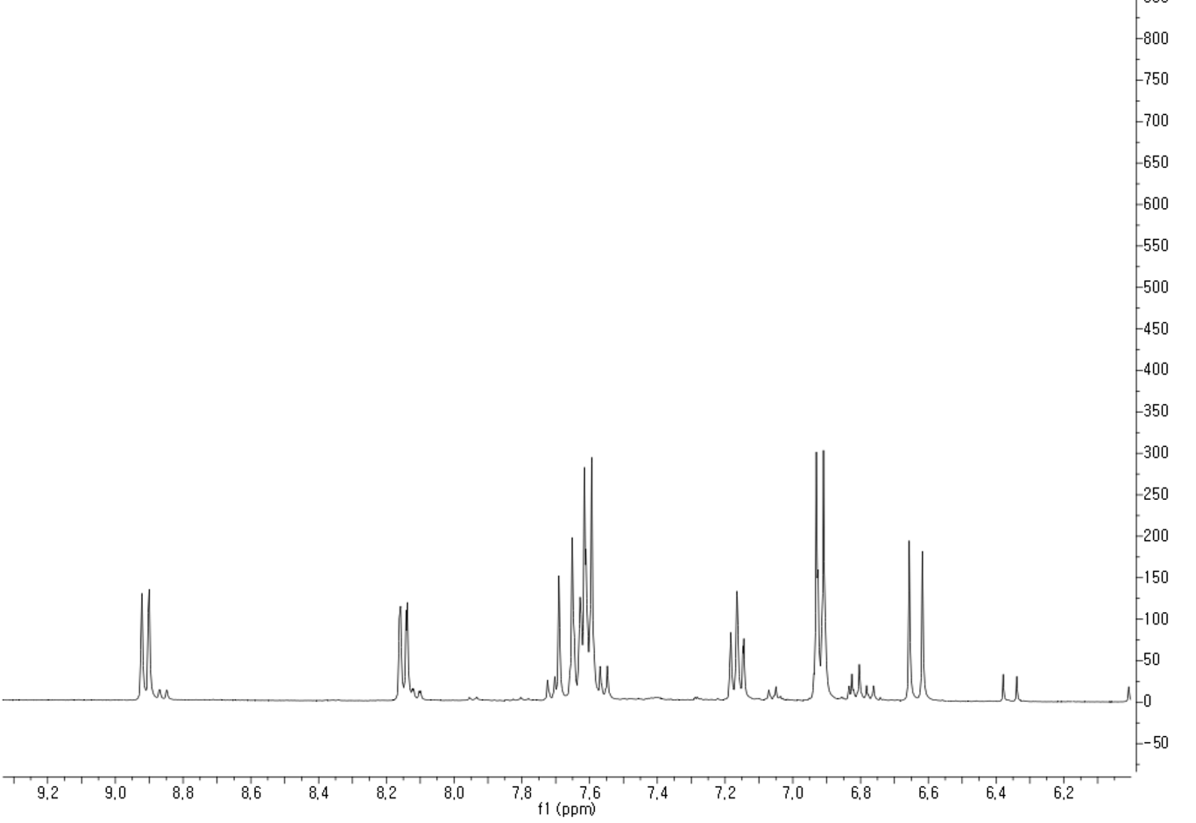


c


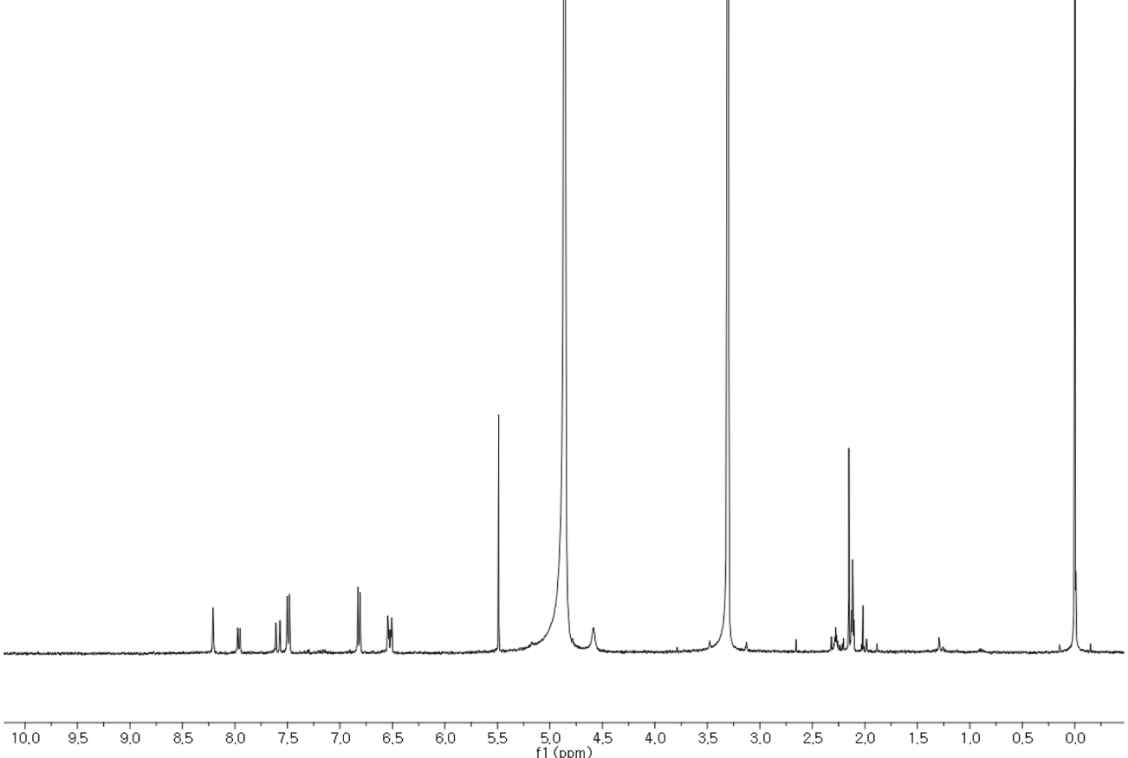


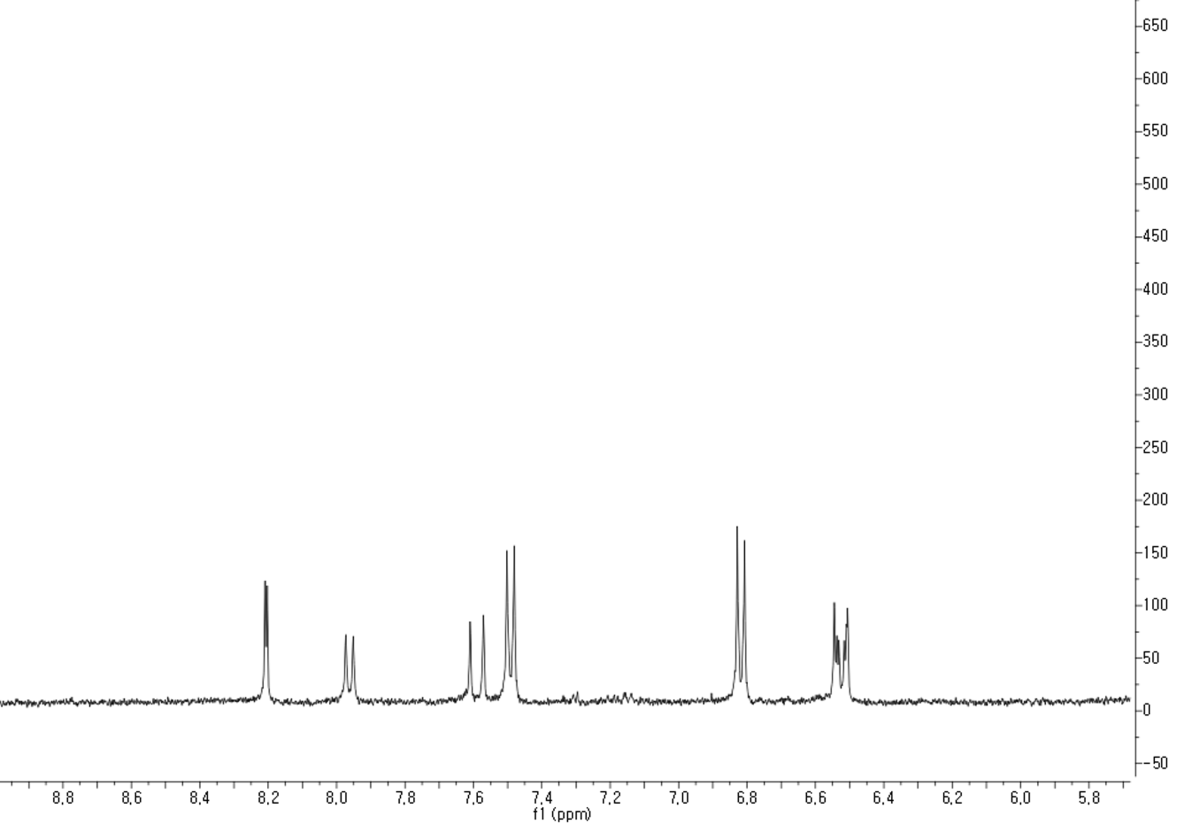


Full and aromatic region of proton NMR spectra of avn A (a), avn D (b), and Avn G (c)

The ^1^H signal intensity of avn A and avn G showed relatively low compared to that of avn D because of huge water signal at around 4.8 ppm existed in the MeOD-*d_6_* which was used as the solvent for avn A and avn G.
